# Supplementary material for: Association of leukocyte composition ratios from blood methylation with cancer mortality outcomes
Source: Commun Med (Lond). 2025 Oct 1;5:411. doi: 10.1038/s43856-025-01132-x (PMC12488966; doi:10.1038/s43856-025-01132-x)
Supplement: Supplementary file 2 — Supplementary Information [file 43856_2025_1132_MOESM2_ESM.pdf]

Supplementary Information of

Association of Leukocyte Composition Ratios from Blood Methylation with Cancer Mortality Outcomes

Authors: Ziwen Fan a, Dominic Edelmann b, Zitong Zhao a, Bruno Christian Köhler c, d, Michael Hoffmeister a, Hermann Brenner a, e, f, g *

Affiliations:

[a] Division of Clinical Epidemiology and Aging Research, German Cancer Research Center (DKFZ); Heidelberg, 69120, Germany.

[b] Division of Biostatistics, German Cancer Research Center (DKFZ); Heidelberg, 69120, Germany

[c] Liver Cancer Center Heidelberg, Heidelberg University Hospital; Heidelberg, 69120, Germany.

[d] Department of Medical Oncology, National Center for Tumor Diseases, Heidelberg University Hospital; Heidelberg, 69120, Germany.

[e] NCT Heidelberg, National Center for Tumor Diseases (NCT), a partnership between DKFZ and University Hospital; Heidelberg, 69120, Germany.

[f] Division of Preventive Oncology, German Cancer Research Center (DKFZ); Heidelberg, 69120, Germany.

[g] German Cancer Consortium (DKTK), German Cancer Research Center (DKFZ); Heidelberg, 69120, Germany.

Correspondence to:

Professor Hermann Brenner, Division of Clinical Epidemiology and Aging Research, German Cancer Research Center (DKFZ), Im Neuenheimer Feld 581, 69120, Heidelberg, Germany, Tel.: +49-6221 421300, Fax: +49-6221 42 1302, E-mail: h.brenner@Dkfz-Heidelberg.de

Contents

Definition of covariates 3

Figure S1. Study selection flow diagram and DNAm data preprocessing. 4

Figure S2. Dose-response relationship for association of LCRs involving lymphocyte with mortality outcomes. 5

Figure S3. Dose-response relationship for association of LCRs involving NK cells with mortality outcomes. 6

Figure S4. Dose-response relationship for association of LCRs involving B cells with mortality outcomes. 7

Figure S5. Dose-response relationship for association of LCRs involving CD4+ T cells with mortality outcomes. 8

Figure S6. Dose-response relationship for association of LCRs involving CD8+ T cells with mortality outcomes. 9

Figure S7. Association of DNAm-derived LCRs with mortality outcomes among males and females. 11

Figure S8. Association of DNAm-derived LCRs with mortality outcomes among younger and older participants. 13

Figure S9. Association of DNAm-derived LCRs with mortality outcomes adjusted for smoking status or smoking pack-years in all subsets. 14

Figure S10. Association of DNAm-derived LCRs with 11-year follow-up mortality outcomes in all subsets. 15

Figure S11. Association of DNAm-derived LCRs with 14-year follow-up mortality outcomes in all subsets. 16

Definition of covariates

The consumption of alcohol was calculated by the following equation: 1 bottle of beer = 11.88 g ethanol, 1 glass of wine = 22.0 g ethanol, 1 shot of liquor = 6.4 g ethanol. Definition of inactive: < 1 h of physical activity/week. Definition of medium or high physical activity: ≥ 2 h of vigorous and ≥ 2 h of light physical activity/week. Definition of low physical activity: all other amounts of activity not categorized as "inactive" or "medium or high". Cardiovascular disease at baseline is a composite variable of either coronary heart disease or history of one or more cardiovascular event (i.e., stroke, myocardial infarction, pulmonary embolism, bypass operation, or dilatation of the coronary vessels). Prevalent diabetes at baseline defined as physician diagnosis or use of glucose lowering drugs or HbA1c ≥6.5% and (fasting glucose ≥126 mg/dL or non-fasting glucose ≥ 200 mg/dL). Prevalent hypertension at baseline defined as physician diagnosis or use of anti-hypertensive drugs or self-reported hypertension plus (systolic blood pressure ≥140 mmHg or diastolic blood pressure ≥90 mmHg).

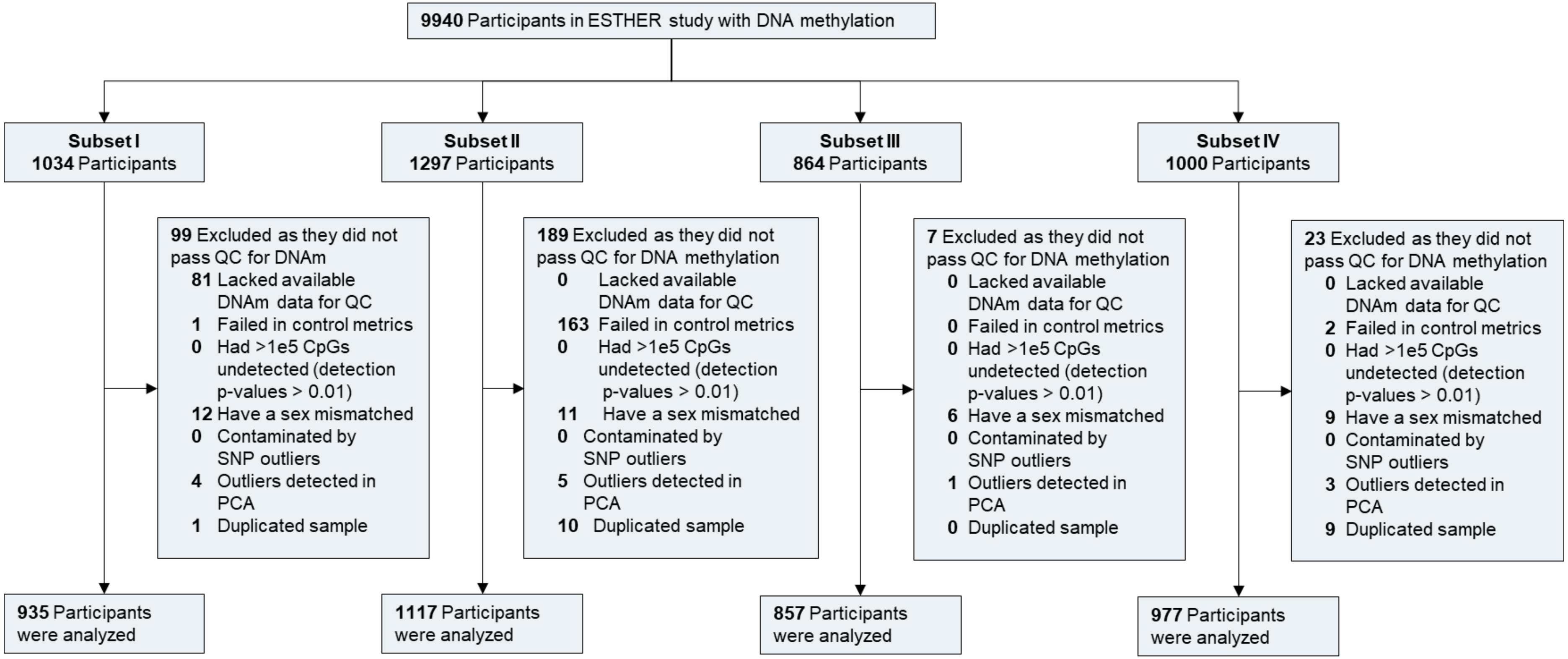

Figure S1. Study selection flow diagram and DNAm data preprocessing.

LCR	Category	All-cause mortality N	Deaths	HR (95% CI)	Cancer-specific mortality N	Deaths	HR (95% CI)	Lung-cancer-specific mortality N	Deaths	HR (95% CI)
**Houseman's algorithm**										
**NLR**	Q1	921	369	**ref**	906	156	**ref**	921	41	**ref**
	Q2	903	370	1.07 (0.85-1.35)	883	140	1.02 (0.76-1.38)	903	37	1.28 (0.65-2.50)
	Q3	918	420	**1.27 (1.06-1.51)**	896	167	1.17 (0.84-1.63)	918	44	1.05 (0.44-2.50)
	Q4	910	484	**1.41 (1.18-1.68)**	892	191	**1.43 (1.00-2.05)**	910	67	1.76 (0.75-4.10)
	Per SD	3652	1643	**1.16 (1.10-1.24)**	3577	654	1.15 (0.97-1.37)	3652	189	1.29 (0.92-1.81)
**LMR**	Q1	911	496	**ref**	889	165	**ref**	911	53	**ref**
	Q2	906	407	**0.75 (0.63-0.88)**	889	186	0.97 (0.68-1.37)	906	55	0.87 (0.50-1.51)
	Q3	919	395	0.84 (0.69-1.01)	899	169	0.93 (0.61-1.42)	919	51	1.13 (0.63-2.04)
	Q4	916	345	**0.65 (0.53-0.80)**	900	134	0.78 (0.48-1.27)	916	30	0.54 (0.29-1.02)
	Per SD	3652	1643	**0.86 (0.80-0.92)**	3577	654	0.87 (0.74-1.03)	3652	189	**0.73 (0.55-0.96)**
**sNMLR**	Q1	912	362	**ref**	896	155	**ref**	912	43	**ref**
	Q2	915	366	1.09 (0.89-1.33)	896	135	0.94 (0.70-1.26)	915	31	0.88 (0.43-1.78)
	Q3	917	435	**1.36 (1.14-1.62)**	897	176	1.21 (0.91-1.62)	917	49	1.24 (0.57-2.68)
	Q4	908	480	**1.41 (1.18-1.69)**	888	188	1.35 (0.87-2.10)	908	66	1.52 (0.61-3.81)
	Per SD	3652	1643	**1.17 (1.10-1.24)**	3577	654	1.15 (0.97-1.37)	3652	189	1.30 (0.93-1.80)
**pNMLR**	Q1	916	343	**ref**	901	135	**ref**	916	28	**ref**
	Q2	922	384	1.21 (0.99-1.48)	901	169	1.19 (0.88-1.62)	922	51	**2.22 (1.14-4.36)**
	Q3	904	423	**1.27 (1.06-1.51)**	882	170	1.24 (0.81-1.89)	904	52	1.77 (0.67-4.70)
	Q4	910	493	**1.60 (1.34-1.91)**	893	180	1.40 (0.91-2.15)	910	58	2.27 (0.99-5.19)
	Per SD	3652	1643	**1.17 (1.10-1.24)**	3577	654	1.14 (0.96-1.35)	3652	189	**1.28 (1.05-1.58)**
**Salas' algorithm**										
**NLR**	Q1	485	188	**ref**	477	79	**ref**	485	23	**ref**
	Q2	493	190	0.99 (0.76-1.28)	482	65	0.90 (0.57-1.42)	493	11	0.59 (0.19-1.84)
	Q3	488	219	1.21 (0.93-1.56)	479	92	1.40 (0.90-2.16)	488	27	1.80 (0.64-5.09)
	Q4	487	247	**1.34 (1.03-1.73)**	476	107	**1.79 (1.20-2.68)**	487	53	**3.87 (1.80-8.32)**
	Per SD	1953	844	**1.15 (1.06-1.26)**	1914	343	**1.28 (1.11-1.49)**	1953	114	**1.75 (1.38-2.21)**
**LMR**	Q1	496	267	**ref**	480	98	**ref**	496	42	**ref**
	Q2	480	191	0.76 (0.48-1.22)	470	84	0.69 (0.42-1.12)	480	33	0.63 (0.30-1.36)
	Q3	482	208	0.87 (0.67-1.12)	477	95	0.70 (0.26-1.88)	482	25	0.63 (0.27-1.46)
	Q4	495	178	**0.73 (0.54-0.97)**	487	66	**0.62 (0.40-0.97)**	495	14	**0.16 (0.06-0.40)**
	Per SD	1953	844	0.92 (0.84-1.01)	1914	343	0.88 (0.75-1.04)	1953	114	**0.53 (0.38-0.75)**
**sNMLR**	Q1	487	189	**ref**	478	81	**ref**	487	23	**ref**
	Q2	492	188	1.00 (0.77-1.29)	484	61	0.81 (0.51-1.28)	492	10	0.79 (0.22-2.83)
	Q3	489	217	1.18 (0.91-1.53)	478	94	1.34 (0.87-2.06)	489	29	2.16 (0.92-5.08)
	Q4	485	250	**1.36 (1.05-1.75)**	474	107	**1.73 (1.16-2.57)**	485	52	**3.81 (1.76-8.24)**
	Per SD	1953	844	**1.16 (1.06-1.26)**	1914	343	**1.28 (1.10-1.48)**	1953	114	**1.76 (1.39-2.23)**
**pNMLR**	Q1	488	184	**ref**	480	71	**ref**	488	19	**ref**
	Q2	493	184	0.90 (0.68-1.18)	487	74	0.60 (0.15-2.33)	493	11	0.65 (0.18-2.32)
	Q3	484	222	1.14 (0.88-1.49)	470	99	1.33 (0.87-2.05)	484	42	**3.44 (1.54-7.69)**
	Q4	488	254	1.29 (0.99-1.68)	477	99	1.49 (0.95-2.32)	488	42	**3.83 (1.55-9.47)**
	Per SD	1953	844	**1.13 (1.04-1.24)**	1914	343	**1.21 (1.04-1.40)**	1953	114	**1.65 (1.29-2.13)**
**LOLIPOP algorithm**										
**NLR**	Q1	919	352	**ref**	905	149	**ref**	919	39	**ref**
	Q2	911	382	1.15 (0.88-1.51)	883	134	1.05 (0.77-1.43)	911	28	0.72 (0.23-2.26)
	Q3	916	423	**1.34 (1.12-1.60)**	902	179	**1.39 (1.04-1.86)**	916	46	1.14 (0.39-3.31)
	Q4	906	486	**1.51 (1.26-1.81)**	887	192	**1.52 (1.03-2.23)**	906	76	**2.35 (1.05-5.24)**
	Per SD	3652	1643	**1.16 (1.09-1.23)**	3577	654	1.17 (0.98-1.38)	3652	189	1.33 (0.99-1.78)
**LMR**	Q1	913	483	**ref**	886	186	**ref**	913	57	**ref**
	Q2	916	415	0.88 (0.70-1.11)	898	174	1.03 (0.68-1.55)	916	48	0.93 (0.51-1.67)
	Q3	918	384	**0.80 (0.66-0.97)**	902	140	0.85 (0.52-1.39)	918	39	0.73 (0.22-2.37)
	Q4	905	361	**0.79 (0.66-0.94)**	891	154	0.99 (0.73-1.33)	905	45	0.80 (0.43-1.50)
	Per SD	3652	1643	0.94 (0.87-1.00)	3577	654	1.01 (0.90-1.12)	3652	189	0.95 (0.74-1.21)
**sNMLR**	Q1	920	344	**ref**	907	147	**ref**	920	37	**ref**
	Q2	913	381	1.18 (0.87-1.60)	884	137	1.03 (0.76-1.38)	913	30	0.86 (0.32-2.32)
	Q3	920	432	**1.39 (1.16-1.67)**	907	179	1.35 (0.97-1.88)	920	47	1.53 (0.74-3.15)
	Q4	899	486	**1.61 (1.34-1.93)**	879	191	1.49 (0.96-2.32)	899	75	**2.55 (1.06-6.10)**
	Per SD	3652	1643	**1.16 (1.10-1.24)**	3577	654	1.16 (0.97-1.38)	3652	189	1.34 (0.99-1.82)
**pNMLR**	Q1	912	360	**ref**	897	151	**ref**	912	43	**ref**
	Q2	926	389	1.04 (0.82-1.32)	912	150	0.95 (0.63-1.42)	926	42	1.14 (0.50-2.59)
	Q3	902	408	1.13 (0.94-1.36)	879	167	0.99 (0.74-1.32)	902	49	1.14 (0.60-2.17)
	Q4	912	486	**1.31 (1.10-1.57)**	889	186	1.10 (0.82-1.48)	912	55	1.41 (0.75-2.67)
	Per SD	3652	1643	**1.12 (1.05-1.19)**	3577	654	1.05 (0.87-1.26)	3652	189	1.21 (0.85-1.72)

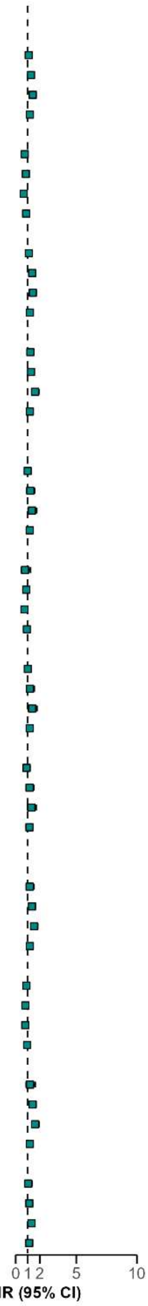

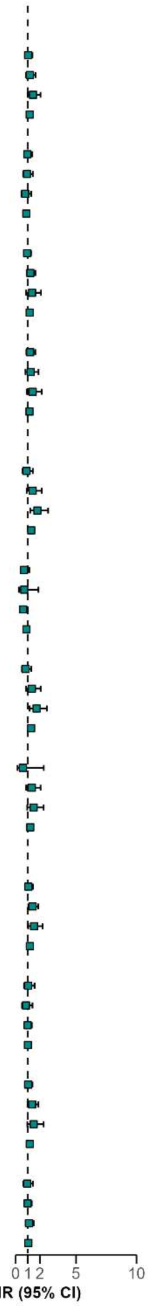

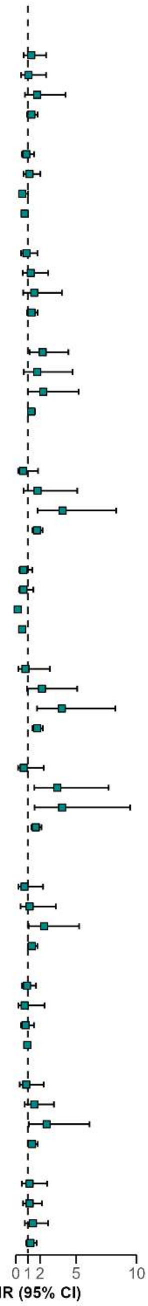

Figure S2. Dose-response relationship for association of LCRs involving lymphocyte with mortality outcomes.

The models were adjusted for age, sex, batch, smoking status, alcohol consumption, body mass index, educational level, physical activity, history of cardiovascular diseases, diabetes, and hypertension. Meta-analysis was performed for subsets I and II using the SAL algorithm and for all subsets using the HOU and LOL algorithms. Values shown in bold indicate statistically significant results. Green squares and error bars depict hazard ratios (HR) along with their 95% confidence intervals (CI).

Abbreviations: LCR, leukocyte composition ratio; HR, hazard ratio; CI, confidence interval; per SD, per standard deviation increase; ref, reference; NLR, neutrophil-to-lymphocyte ratio; sNMLR, ratio of sum of neutrophil and monocytes divided by lymphocyte; pNMLR, ratio of product of neutrophil and monocytes divided by lymphocyte; LMR, lymphocyte-to-monocyte ratio.

LCR	Category	All-cause mortality			Cancer-specific mortality			Lung-cancer-specific mortality		
		N	Deaths	HR (95% CI)	N	Deaths	HR (95% CI)	N	Deaths	HR (95% CI)
**Houseman's algorithm**										
**NNKR**	Q1	909	421	**ref**	893	160	**ref**	909	43	**ref**
	Q2	898	402	1.10 (0.90-1.34)	871	147	1.09 (0.82-1.46)	898	32	0.82 (0.42-1.58)
	Q3	928	413	**1.22 (1.02-1.45)**	916	183	1.35 (0.98-1.87)	928	57	1.51 (0.81-2.82)
	Q4	917	407	**1.36 (1.14-1.63)**	897	164	1.26 (0.85-1.85)	917	57	1.44 (0.77-2.69)
	Per SD	3652	1643	**1.12 (1.04-1.19)**	3577	654	1.08 (0.90-1.28)	3652	189	1.16 (0.95-1.43)
**NKMR**	Q1	936	428	**ref**	916	172	**ref**	936	53	**ref**
	Q2	898	425	1.04 (0.87-1.23)	876	160	1.00 (0.74-1.34)	898	49	0.94 (0.53-1.66)
	Q3	910	390	**0.73 (0.61-0.87)**	894	170	0.83 (0.53-1.29)	910	54	0.64 (0.30-1.34)
	Q4	908	400	**0.70 (0.58-0.83)**	891	152	0.75 (0.46-1.21)	908	33	0.47 (0.19-1.19)
	Per SD	3652	1643	**0.89 (0.81-0.99)**	3577	654	0.89 (0.75-1.06)	3652	189	**0.76 (0.59-0.99)**
**sNMNKR**	Q1	907	416	**ref**	891	154	**ref**	907	43	**ref**
	Q2	901	403	1.16 (0.91-1.46)	874	158	1.23 (0.93-1.64)	901	35	0.89 (0.46-1.69)
	Q3	921	415	**1.28 (1.08-1.53)**	909	176	**1.42 (1.08-1.89)**	921	53	1.46 (0.84-2.54)
	Q4	923	409	**1.39 (1.16-1.66)**	903	166	1.31 (0.89-1.94)	923	58	1.42 (0.69-2.93)
	Per SD	3652	1643	**1.12 (1.04-1.20)**	3577	654	1.08 (0.90-1.29)	3652	189	1.17 (0.96-1.43)
**pNMNKR**	Q1	902	399	**ref**	884	149	**ref**	902	36	**ref**
	Q2	922	392	0.97 (0.82-1.16)	906	169	1.02 (0.77-1.37)	922	48	1.10 (0.34-3.52)
	Q3	896	422	**1.37 (1.04-1.81)**	876	167	1.31 (0.95-1.81)	896	49	1.68 (0.79-3.56)
	Q4	932	430	**1.33 (1.11-1.59)**	911	169	1.21 (0.80-1.85)	932	56	1.79 (0.82-3.91)
	Per SD	3652	1643	**1.14 (1.07-1.21)**	3577	654	1.10 (0.91-1.33)	3652	189	1.19 (0.97-1.46)
**Salas' algorithm**										
**NNKR**	Q1	475	217	**ref**	466	77	**ref**	475	23	**ref**
	Q2	482	201	1.03 (0.80-1.33)	472	79	1.02 (0.54-1.94)	482	14	0.61 (0.18-2.11)
	Q3	494	217	1.22 (0.85-1.76)	484	91	**1.79 (1.20-2.67)**	494	37	**2.61 (1.26-5.41)**
	Q4	502	209	1.17 (0.90-1.53)	492	96	1.36 (0.80-2.30)	502	40	1.85 (0.81-4.24)
	Per SD	1953	844	1.09 (0.99-1.20)	1914	343	1.13 (0.98-1.31)	1953	114	**1.48 (1.14-1.94)**
**NKMR**	Q1	506	220	**ref**	497	85	**ref**	506	38	**ref**
	Q2	497	208	0.86 (0.66-1.11)	483	84	1.11 (0.73-1.69)	497	28	0.80 (0.37-1.72)
	Q3	476	203	**0.75 (0.58-0.97)**	470	99	0.99 (0.45-2.20)	476	32	0.44 (0.05-3.94)
	Q4	474	213	0.83 (0.64-1.09)	464	75	0.91 (0.58-1.41)	474	16	**0.32 (0.14-0.75)**
	Per SD	1953	844	0.96 (0.86-1.07)	1914	343	0.89 (0.77-1.04)	1953	114	**0.65 (0.48-0.88)**
**sNMNKR**	Q1	473	215	**ref**	463	78	**ref**	473	23	**ref**
	Q2	484	205	1.02 (0.74-1.42)	474	80	1.11 (0.69-1.79)	484	16	0.66 (0.20-2.13)
	Q3	496	221	1.27 (0.89-1.82)	487	95	**1.74 (1.16-2.60)**	496	38	**2.69 (1.31-5.53)**
	Q4	500	203	1.14 (0.88-1.49)	490	90	1.34 (0.87-2.07)	500	37	1.75 (0.77-3.94)
	Per SD	1953	844	1.09 (0.99-1.19)	1914	343	1.12 (0.97-1.30)	1953	114	**1.48 (1.14-1.93)**
**pNMNKR**	Q1	478	209	**ref**	469	77	**ref**	478	16	**ref**
	Q2	484	203	0.91 (0.70-1.18)	479	96	0.95 (0.38-2.38)	484	28	**3.98 (1.46-10.82)**
	Q3	487	205	1.21 (0.93-1.57)	471	79	1.26 (0.82-1.95)	487	34	**4.12 (1.53-11.07)**
	Q4	504	227	1.22 (0.94-1.59)	495	91	1.26 (0.82-1.94)	504	36	**3.33 (1.45-7.67)**
	Per SD	1953	844	**1.10 (1.01-1.21)**	1914	343	1.12 (0.97-1.29)	1953	114	**1.49 (1.17-1.91)**

Figure S3. Dose-response relationship for association of LCRs involving NK cells with mortality outcomes.

The models were adjusted for age, sex, batch, smoking status, alcohol consumption, body mass index, educational level, physical activity, history of cardiovascular diseases, diabetes, and hypertension. Meta-analysis was performed for subsets I and II using the SAL algorithm and for all subsets using the HOU and LOL algorithms. Values shown in bold indicate statistically significant results. Green squares and error bars depict hazard ratios (HR) along with their 95% confidence intervals (CI).

Abbreviations: LCR, leukocyte composition ratio; HR, hazard ratio; CI, confidence interval; per SD, per standard deviation increase; ref, reference; NK, natural killer cells; NNKR, neutrophil-to-NK ratio; sNMNKR, ratio of sum of neutrophil and monocytes divided by NK; pNMNKR, ratio of product of neutrophil and monocytes divided by NK; NKMR, NK-to-monocyte ratio.

LCR	Category	All-cause mortality			Cancer-specific mortality			Lung-cancer-specific mortality		
		N	Deaths	HR (95% CI)	N	Deaths	HR (95% CI)	N	Deaths	HR (95% CI)
**Houseman's algorithm**										
**NBR**	Q1	936	359	**ref**	921	162	**ref**	936	52	**ref**
	Q2	904	359	1.05 (0.87-1.26)	890	149	0.94 (0.64-1.38)	904	35	0.66 (0.26-1.65)
	Q3	904	423	**1.25 (1.04-1.50)**	886	169	1.12 (0.85-1.49)	904	50	1.19 (0.67-2.11)
	Q4	908	502	**1.47 (1.23-1.76)**	880	174	1.26 (0.94-1.68)	908	52	1.13 (0.43-2.97)
	Per SD	3652	1643	**1.14 (1.06-1.23)**	3577	654	**1.11 (1.00-1.23)**	3652	189	1.04 (0.72-1.50)
**BMR**	Q1	906	499	**ref**	879	167	**ref**	906	47	**ref**
	Q2	918	444	0.93 (0.79-1.10)	903	191	0.99 (0.75-1.29)	918	61	1.11 (0.64-1.94)
	Q3	904	357	**0.75 (0.63-0.90)**	888	152	0.78 (0.58-1.05)	904	40	0.84 (0.45-1.56)
	Q4	924	343	**0.69 (0.57-0.84)**	907	144	0.78 (0.58-1.05)	924	41	0.81 (0.44-1.50)
	Per SD	3652	1643	**0.88 (0.83-0.94)**	3577	654	0.93 (0.84-1.04)	3652	189	0.88 (0.71-1.09)
**sNMBR**	Q1	932	349	**ref**	916	163	**ref**	932	48	**ref**
	Q2	914	368	1.12 (0.87-1.44)	902	147	0.89 (0.64-1.25)	914	37	0.79 (0.38-1.64)
	Q3	899	422	**1.33 (1.11-1.60)**	880	169	1.12 (0.84-1.48)	899	51	1.31 (0.74-2.32)
	Q4	907	504	**1.54 (1.28-1.85)**	879	175	1.26 (0.95-1.68)	907	53	1.23 (0.48-3.14)
	Per SD	3652	1643	**1.14 (1.06-1.23)**	3577	654	1.11 (1.00-1.22)	3652	189	1.04 (0.72-1.48)
**pNMBR**	Q1	927	336	**ref**	909	145	**ref**	927	36	**ref**
	Q2	910	369	**1.22 (1.01-1.47)**	897	157	1.05 (0.78-1.42)	910	48	1.45 (0.77-2.70)
	Q3	913	445	**1.45 (1.21-1.74)**	893	180	1.23 (0.92-1.63)	913	54	1.49 (0.81-2.72)
	Q4	902	493	**1.49 (1.22-1.82)**	878	172	1.31 (0.97-1.76)	902	51	1.46 (0.76-2.80)
	Per SD	3652	1643	**1.14 (1.06-1.22)**	3577	654	**1.11 (1.01-1.23)**	3652	189	1.08 (0.81-1.43)
**Salas' algorithm**										
**NBR**	Q1	498	184	**ref**	493	81	**ref**	498	22	**ref**
	Q2	490	188	0.90 (0.68-1.18)	484	81	0.83 (0.54-1.29)	490	25	1.41 (0.43-4.62)
	Q3	473	209	1.14 (0.87-1.50)	462	92	1.21 (0.80-1.85)	473	30	2.16 (0.92-5.08)
	Q4	492	263	**1.38 (1.07-1.79)**	475	89	1.11 (0.55-2.25)	492	37	1.75 (0.43-7.14)
	Per SD	1953	844	**1.20 (1.10-1.31)**	1914	343	1.15 (0.87-1.54)	1953	114	1.26 (0.80-2.00)
**BMR**	Q1	495	258	**ref**	479	86	**ref**	495	29	**ref**
	Q2	478	232	0.94 (0.74-1.19)	463	99	1.05 (0.54-2.04)	478	44	1.23 (0.58-2.64)
	Q3	482	182	0.79 (0.54-1.15)	480	84	0.98 (0.65-1.50)	482	23	0.91 (0.34-2.46)
	Q4	498	172	**0.73 (0.56-0.95)**	492	74	0.81 (0.51-1.29)	498	18	0.57 (0.22-1.48)
	Per SD	1953	844	**0.86 (0.78-0.95)**	1914	343	0.94 (0.77-1.16)	1953	114	0.79 (0.53-1.18)
**sNMBR**	Q1	498	180	**ref**	492	79	**ref**	498	22	**ref**
	Q2	489	188	0.97 (0.74-1.27)	485	81	0.88 (0.55-1.40)	489	24	1.40 (0.38-5.14)
	Q3	473	209	1.15 (0.88-1.51)	461	92	1.19 (0.78-1.83)	473	31	2.19 (0.95-5.09)
	Q4	493	267	**1.46 (1.13-1.89)**	476	91	1.16 (0.55-2.42)	493	37	1.90 (0.63-5.72)
	Per SD	1953	844	**1.20 (1.10-1.31)**	1914	343	1.15 (0.87-1.52)	1953	114	1.26 (0.81-1.97)
**pNMBR**	Q1	496	175	**ref**	490	79	**ref**	496	17	**ref**
	Q2	488	181	1.00 (0.76-1.32)	485	76	0.92 (0.59-1.43)	488	21	1.49 (0.60-3.68)
	Q3	476	231	1.23 (0.94-1.62)	464	99	1.10 (0.71-1.70)	476	44	**2.88 (1.23-6.73)**
	Q4	493	257	**1.36 (1.04-1.78)**	475	89	1.27 (0.82-1.96)	493	32	2.23 (0.85-5.85)
	Per SD	1953	844	**1.17 (1.07-1.28)**	1914	343	1.15 (0.92-1.43)	1953	114	**1.35 (1.00-1.82)**

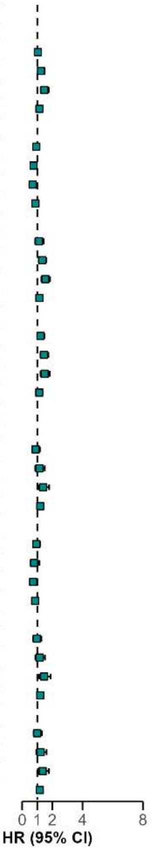

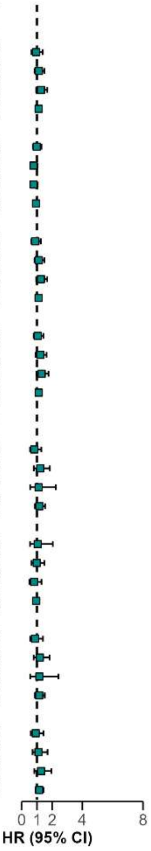

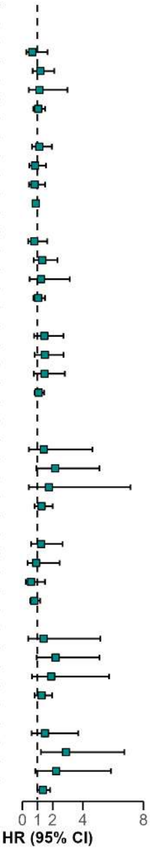

Figure S4. Dose-response relationship for association of LCRs involving B cells with mortality outcomes.

The models were adjusted for age, sex, batch, smoking status, alcohol consumption, body mass index, educational level, physical activity, history of cardiovascular diseases, diabetes, and hypertension. Meta-analysis was performed for subsets I and II using the SAL algorithm and for all subsets using the HOU and LOL algorithms. Values shown in bold indicate statistically significant results. Green squares and error bars depict hazard ratios (HR) along with their 95% confidence intervals (CI).

Abbreviations: LCR, leukocyte composition ratio; HR, hazard ratio; CI, confidence interval; per SD, per standard deviation increase; ref, reference; B, B cells; NBR, neutrophil-to-B ratio; sNMBR, ratio of sum of neutrophil and monocytes divided by B; pNMBR, ratio of product of neutrophil and monocytes divided by B; BMR, B-to-monocyte ratio.

LCR	Category	All-cause mortality N	All-cause mortality Deaths	All-cause mortality HR (95% CI)	Cancer-specific mortality N	Cancer-specific mortality Deaths	Cancer-specific mortality HR (95% CI)	Lung-cancer-specific mortality N	Lung-cancer-specific mortality Deaths	Lung-cancer-specific mortality HR (95% CI)
**Houseman's algorithm**										
**NCD4TR**	Q1	927	341	**ref**	910	142	**ref**	927	37	**ref**
	Q2	919	414	**1.33 (1.11-1.59)**	904	190	**1.64 (1.20-2.24)**	919	47	**2.15 (1.12-4.14)**
	Q3	920	413	1.17 (0.90-1.52)	894	147	1.08 (0.80-1.47)	920	44	1.23 (0.54-2.80)
	Q4	886	475	**1.44 (1.16-1.79)**	869	175	**1.53 (1.15-2.04)**	886	61	**2.36 (1.27-4.37)**
	Per SD	3652	1643	**1.13 (1.07-1.21)**	3577	654	**1.13 (1.03-1.25)**	3652	189	**1.25 (1.02-1.54)**
**CD4TMR**	Q1	895	478	**ref**	876	166	**ref**	895	53	**ref**
	Q2	925	439	0.93 (0.77-1.12)	904	191	1.14 (0.87-1.51)	925	58	1.27 (0.73-2.20)
	Q3	903	394	0.96 (0.81-1.15)	887	164	1.00 (0.75-1.34)	903	50	1.04 (0.58-1.85)
	Q4	929	332	**0.72 (0.60-0.87)**	910	133	0.80 (0.59-1.09)	929	28	**0.51 (0.26-1.00)**
	Per SD	3652	1643	**0.87 (0.80-0.94)**	3577	654	0.89 (0.79-1.00)	3652	189	**0.76 (0.61-0.96)**
**sNMCD4TR**	Q1	928	338	**ref**	911	133	**ref**	928	35	**ref**
	Q2	919	406	**1.33 (1.10-1.60)**	904	195	**1.69 (1.03-2.76)**	919	50	**2.77 (1.51-5.08)**
	Q3	914	422	1.21 (0.93-1.57)	884	151	1.23 (0.90-1.67)	914	41	1.21 (0.60-2.44)
	Q4	891	477	**1.45 (1.15-1.83)**	878	175	**1.59 (1.16-2.19)**	891	63	**2.68 (1.47-4.90)**
	Per SD	3652	1643	**1.14 (1.07-1.21)**	3577	654	**1.13 (1.03-1.25)**	3652	189	**1.27 (1.04-1.54)**
**pNMCD4TR**	Q1	924	327	**ref**	906	133	**ref**	924	27	**ref**
	Q2	913	396	**1.31 (1.09-1.57)**	898	173	1.30 (0.91-1.84)	913	51	**2.15 (1.15-4.03)**
	Q3	919	439	**1.33 (1.11-1.59)**	892	170	1.29 (0.84-1.99)	919	50	1.93 (0.92-4.03)
	Q4	896	481	**1.44 (1.20-1.72)**	881	178	1.37 (0.82-2.29)	896	61	**2.14 (1.14-4.05)**
	Per SD	3652	1643	**1.13 (1.05-1.21)**	3577	654	**1.12 (1.01-1.23)**	3652	189	**1.24 (1.02-1.51)**
**Salas' algorithm**										
**NCD4TR**	Q1	494	174	**ref**	485	71	**ref**	494	21	**ref**
	Q2	484	198	1.14 (0.88-1.49)	475	85	1.31 (0.71-2.43)	484	19	2.25 (0.97-5.22)
	Q3	512	219	0.96 (0.73-1.26)	501	93	1.14 (0.74-1.76)	512	31	1.72 (0.74-3.99)
	Q4	463	253	1.27 (0.97-1.67)	453	94	**1.63 (1.06-2.49)**	463	43	**3.36 (1.51-7.47)**
	Per SD	1953	844	1.08 (0.98-1.18)	1914	343	1.15 (1.00-1.34)	1953	114	**1.41 (1.09-1.84)**
**CD4TMR**	Q1	481	255	**ref**	468	98	**ref**	481	43	**ref**
	Q2	487	220	0.92 (0.72-1.17)	476	88	0.79 (0.44-1.42)	487	23	0.34 (0.06-1.88)
	Q3	486	202	1.07 (0.82-1.38)	478	91	0.99 (0.65-1.49)	486	34	1.06 (0.52-2.15)
	Q4	499	167	0.86 (0.65-1.14)	492	66	0.76 (0.47-1.21)	499	14	**0.34 (0.13-0.87)**
	Per SD	1953	844	0.95 (0.86-1.04)	1914	343	0.94 (0.79-1.10)	1953	114	**0.70 (0.50-0.98)**
**sNMCD4TR**	Q1	492	169	**ref**	484	66	**ref**	492	17	**ref**
	Q2	487	202	1.12 (0.86-1.46)	477	94	1.42 (0.71-2.81)	487	23	**2.71 (1.16-6.33)**
	Q3	512	222	1.04 (0.80-1.37)	502	93	1.20 (0.77-1.86)	512	33	1.90 (0.51-7.12)
	Q4	462	251	1.21 (0.92-1.59)	451	90	**1.56 (1.01-2.41)**	462	41	**3.33 (1.44-7.73)**
	Per SD	1953	844	1.07 (0.98-1.18)	1914	343	1.15 (0.99-1.33)	1953	114	**1.40 (1.08-1.82)**
**pNMCD4TR**	Q1	495	161	**ref**	488	69	**ref**	495	14	**ref**
	Q2	491	212	1.32 (0.98-1.79)	480	86	1.25 (0.80-1.96)	491	24	**2.64 (1.05-6.63)**
	Q3	490	208	1.07 (0.81-1.42)	480	88	1.02 (0.65-1.60)	490	35	1.67 (0.18-15.16)
	Q4	477	263	1.29 (0.97-1.70)	466	100	1.40 (0.88-2.22)	477	41	**3.51 (1.29-9.60)**
	Per SD	1953	844	1.07 (0.97-1.18)	1914	343	1.12 (0.96-1.31)	1953	114	**1.38 (1.04-1.83)**

Figure S5. Dose-response relationship for association of LCRs involving CD4+ T cells with mortality outcomes.

The models were adjusted for age, sex, batch, smoking status, alcohol consumption, body mass index, educational level, physical activity, history of cardiovascular diseases, diabetes, and hypertension. Meta-analysis was performed for subsets I and II using the SAL algorithm and for all subsets using the HOU and LOL algorithms. Values shown in bold indicate statistically significant results. Green squares and error bars depict hazard ratios (HR) along with their 95% confidence intervals (CI).

Abbreviations: LCR, leukocyte composition ratio; HR, hazard ratio; CI, confidence interval; per SD, per standard deviation increase; ref, reference; CD4T, $CD4^+$ T cells; NCD4TR, neutrophil-to-CD4T ratio; sNMCD4TR, ratio of sum of neutrophil and monocytes divided by CD4T; pNMCD4TR, ratio of product of neutrophil and monocytes divided by CD4T; CD4TMR, CD4T-to-monocyte ratio.

LCR	Category	All-cause mortality N	Deaths	HR (95% CI)	Cancer-specific mortality N	Deaths	HR (95% CI)	Lung-cancer-specific mortality N	Deaths	HR (95% CI)
**Houseman's algorithm**										
**NCD8TR**	Q1	907	369	**ref**	885	151	**ref**	907	45	**ref**
	Q2	911	376	1.03 (0.86-1.23)	897	152	1.03 (0.73-1.44)	911	33	0.62 (0.33-1.17)
	Q3	900	386	1.04 (0.87-1.25)	886	150	0.95 (0.51-1.75)	900	55	0.88 (0.30-2.59)
	Q4	934	512	1.17 (0.90-1.51)	909	201	1.25 (0.68-2.29)	934	56	0.80 (0.12-5.25)
	Per SD	3652	1643	1.05 (0.98-1.11)	3577	654	1.07 (0.94-1.22)	3652	189	0.99 (0.60-1.62)
**CD8TMR**	Q1	945	520	**ref**	920	205	**ref**	945	57	**ref**
	Q2	893	402	0.94 (0.79-1.11)	879	146	0.78 (0.58-1.05)	893	54	1.05 (0.21-5.26)
	Q3	916	373	0.89 (0.75-1.07)	901	167	0.92 (0.69-1.24)	916	41	0.95 (0.34-2.62)
	Q4	898	348	**0.82 (0.69-0.99)**	877	136	0.78 (0.50-1.21)	898	37	1.54 (0.24-9.84)
	Per SD	3652	1643	**0.94 (0.88-1.00)**	3577	654	0.94 (0.79-1.11)	3652	189	0.81 (0.44-1.49)
**sNMCD8TR**	Q1	909	363	**ref**	887	142	**ref**	909	43	**ref**
	Q2	909	378	1.02 (0.85-1.22)	895	159	1.16 (0.80-1.67)	909	31	0.59 (0.31-1.12)
	Q3	899	392	1.12 (0.93-1.35)	885	154	1.12 (0.59-2.12)	899	57	1.14 (0.44-2.92)
	Q4	935	510	1.18 (0.92-1.52)	910	199	1.35 (0.72-2.53)	935	58	0.88 (0.14-5.56)
	Per SD	3652	1643	1.05 (0.98-1.11)	3577	654	1.07 (0.94-1.22)	3652	189	0.99 (0.60-1.64)
**pNMCD8TR**	Q1	907	348	**ref**	887	141	**ref**	907	41	**ref**
	Q2	907	363	1.01 (0.84-1.22)	891	147	0.98 (0.73-1.34)	907	27	**0.30 (0.12-0.75)**
	Q3	896	411	1.20 (1.00-1.43)	882	159	1.11 (0.81-1.53)	896	64	1.41 (0.78-2.55)
	Q4	942	521	**1.24 (1.03-1.48)**	917	207	1.32 (0.84-2.07)	942	57	0.54 (0.06-4.96)
	Per SD	3652	1643	**1.07 (1.00-1.13)**	3577	654	1.09 (0.95-1.25)	3652	189	1.01 (0.60-1.71)
**Salas' algorithm**										
**NCD8TR**	Q1	477	206	**ref**	467	83	**ref**	477	22	**ref**
	Q2	492	208	1.19 (0.92-1.54)	481	74	1.17 (0.75-1.82)	492	25	1.42 (0.62-3.24)
	Q3	491	202	1.09 (0.84-1.41)	481	88	1.37 (0.89-2.10)	491	31	2.29 (0.98-5.35)
	Q4	493	228	**1.31 (1.01-1.70)**	485	98	**1.69 (1.12-2.54)**	493	36	**3.39 (1.48-7.77)**
	Per SD	1953	844	**1.12 (1.02-1.22)**	1914	343	**1.19 (1.04-1.37)**	1953	114	**1.59 (1.22-2.07)**
**CD8TMR**	Q1	491	238	**ref**	481	94	**ref**	491	41	**ref**
	Q2	495	202	0.84 (0.45-1.56)	485	87	0.76 (0.47-1.25)	495	26	0.50 (0.13-1.96)
	Q3	493	194	**0.77 (0.60-0.99)**	487	86	0.81 (0.54-1.21)	493	23	0.36 (0.10-1.30)
	Q4	474	210	0.85 (0.66-1.09)	461	76	0.68 (0.44-1.05)	474	24	**0.33 (0.14-0.78)**
	Per SD	1953	844	0.94 (0.86-1.03)	1914	343	0.91 (0.77-1.06)	1953	114	**0.61 (0.42-0.89)**
**sNMCD8TR**	Q1	481	211	**ref**	471	85	**ref**	481	26	**ref**
	Q2	487	197	1.13 (0.87-1.47)	479	76	1.19 (0.76-1.86)	487	21	0.77 (0.31-1.93)
	Q3	493	206	1.14 (0.88-1.48)	480	83	1.26 (0.81-1.95)	493	28	1.32 (0.55-3.15)
	Q4	492	230	1.29 (1.00-1.67)	484	99	**1.65 (1.09-2.49)**	492	39	**2.59 (1.17-5.71)**
	Per SD	1953	844	**1.12 (1.03-1.22)**	1914	343	**1.19 (1.03-1.36)**	1953	114	**1.58 (1.21-2.06)**
**pNMCD8TR**	Q1	481	204	**ref**	470	74	**ref**	481	20	**ref**
	Q2	489	187	1.03 (0.76-1.41)	481	81	1.31 (0.84-2.04)	489	27	1.87 (0.84-4.17)
	Q3	489	217	1.15 (0.89-1.48)	480	97	1.44 (0.93-2.23)	489	29	**2.36 (1.04-5.32)**
	Q4	494	236	1.24 (0.88-1.73)	483	91	**1.60 (1.04-2.46)**	494	38	**4.07 (1.72-9.65)**
	Per SD	1953	844	**1.12 (1.03-1.22)**	1914	343	**1.16 (1.01-1.33)**	1953	114	**1.57 (1.20-2.04)**

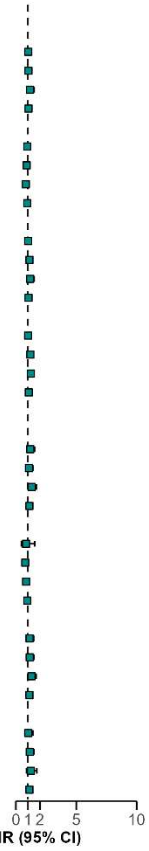

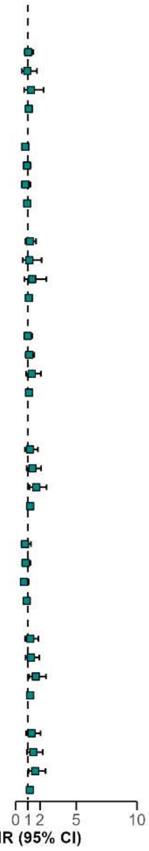

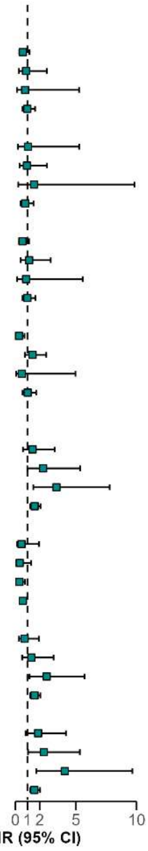

Figure S6. Dose-response relationship for association of LCRs involving CD8+ T cells with mortality outcomes.

The models were adjusted for age, sex, batch, smoking status, alcohol consumption, body mass index, educational level, physical activity, history of cardiovascular diseases, diabetes, and hypertension. Meta-analysis was performed for subsets I and II using the SAL algorithm and for all subsets using the HOU and LOL algorithms. Values shown in bold indicate statistically significant results. Green squares and error bars depict hazard ratios (HR) along with their 95% confidence intervals (CI).

Abbreviations: LCR, leukocyte composition ratio; HR, hazard ratio; CI, confidence interval; per SD, per standard deviation increase; ref, reference; CD8T, $CD8^+$ T cells; NCD8TR, neutrophil-to-CD8T ratio; sNMCD8TR, ratio of sum of neutrophil and monocytes divided by CD8T; pNMCD8TR, ratio of product of neutrophil and monocytes divided by CD8T; CD8TMR, CD8T-to-monocyte ratio.

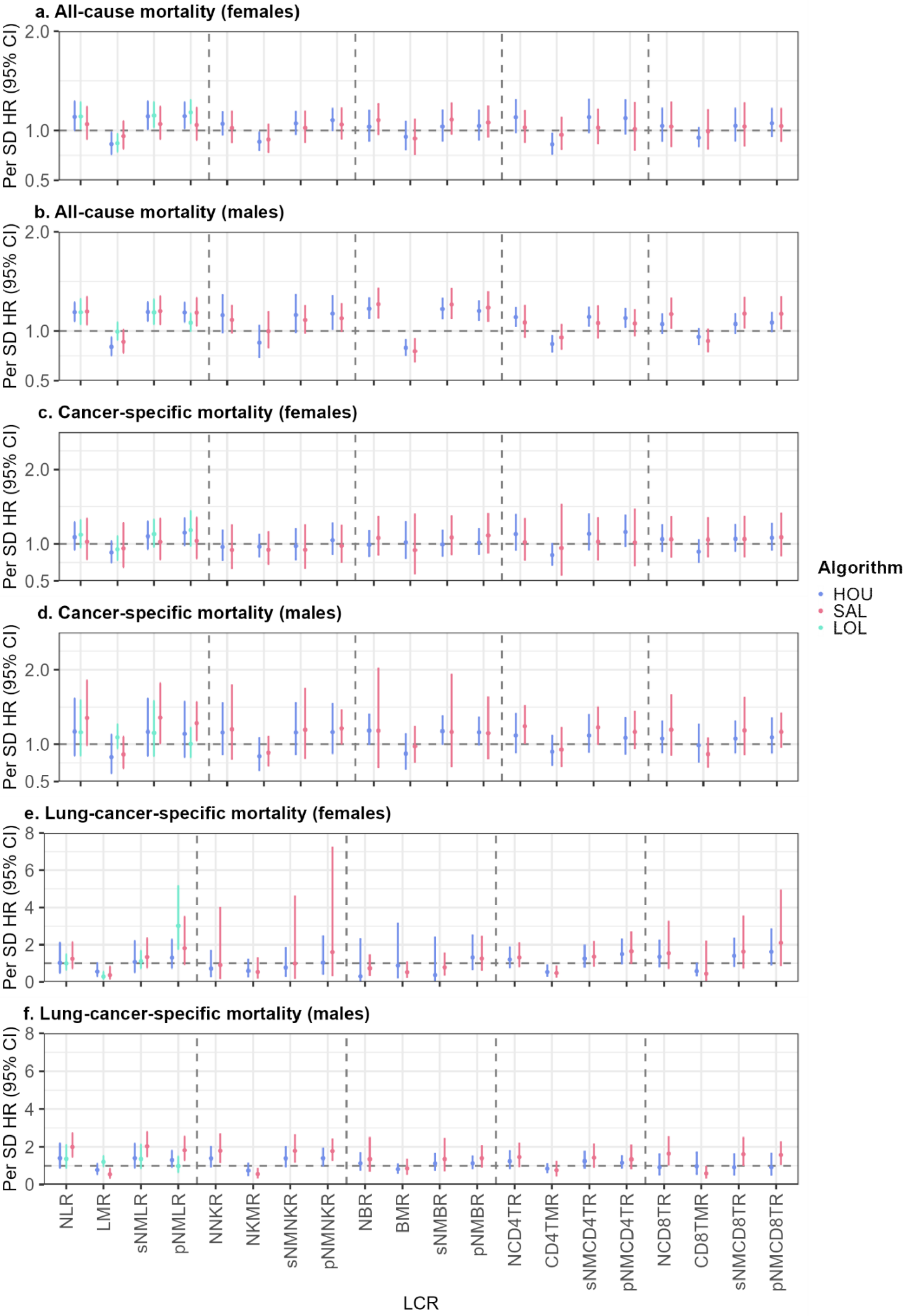

a. All-cause mortality (females)
b. All-cause mortality (males)
c. Cancer-specific mortality (females)
d. Cancer-specific mortality (males)
e. Lung-cancer-specific mortality (females)
f. Lung-cancer-specific mortality (males)
Per SD HR (95% CI)
0.5
1.0
2.0
0
2
4
6
8
NLR
LMR
sNMLR
pNMLR
NNKR
NKMR
sNMNKR
pNMNKR
NBR
BMR
sNMBR
pNMBR
NCD4TR
CD4TMR
sNMCD4TR
pNMCD4TR
NCD8TR
CD8TMR
sNMCD8TR
pNMCD8TR
LCR
Algorithm
HOU
SAL
LOL

Figure S7. Association of DNAm-derived LCRs with mortality outcomes among males and females.

The models were adjusted for age, batch, smoking status, alcohol consumption, body mass index, educational level, physical activity, history of cardiovascular diseases, diabetes, and hypertension. Meta-analysis was performed for subsets I and II using the SAL algorithm and for all subsets using the HOU and LOL algorithms. Dot and error bars depict hazard ratios (HR) along with their 95% confidence intervals (CI).

Abbreviations: LCR, leukocyte composition ratio; HR, hazard ratio; CI, confidence interval; Per SD, per standard deviation increase; HOU, Housman's algorithm; SAL, Salas' algorithm; LOL, LOLIPOP algorithm; NLR, neutrophil-to-lymphocyte ratio; sNMLR, ratio of sum of neutrophil and monocytes divided by lymphocyte; pNMLR, ratio of product of neutrophil and monocytes divided by lymphocyte; LMR, lymphocyte-to-monocyte ratio; B, B cell; CD4T, $CD4^+$ T cell; CD8T, $CD8^+$ T cell; NK, natural killer cell.

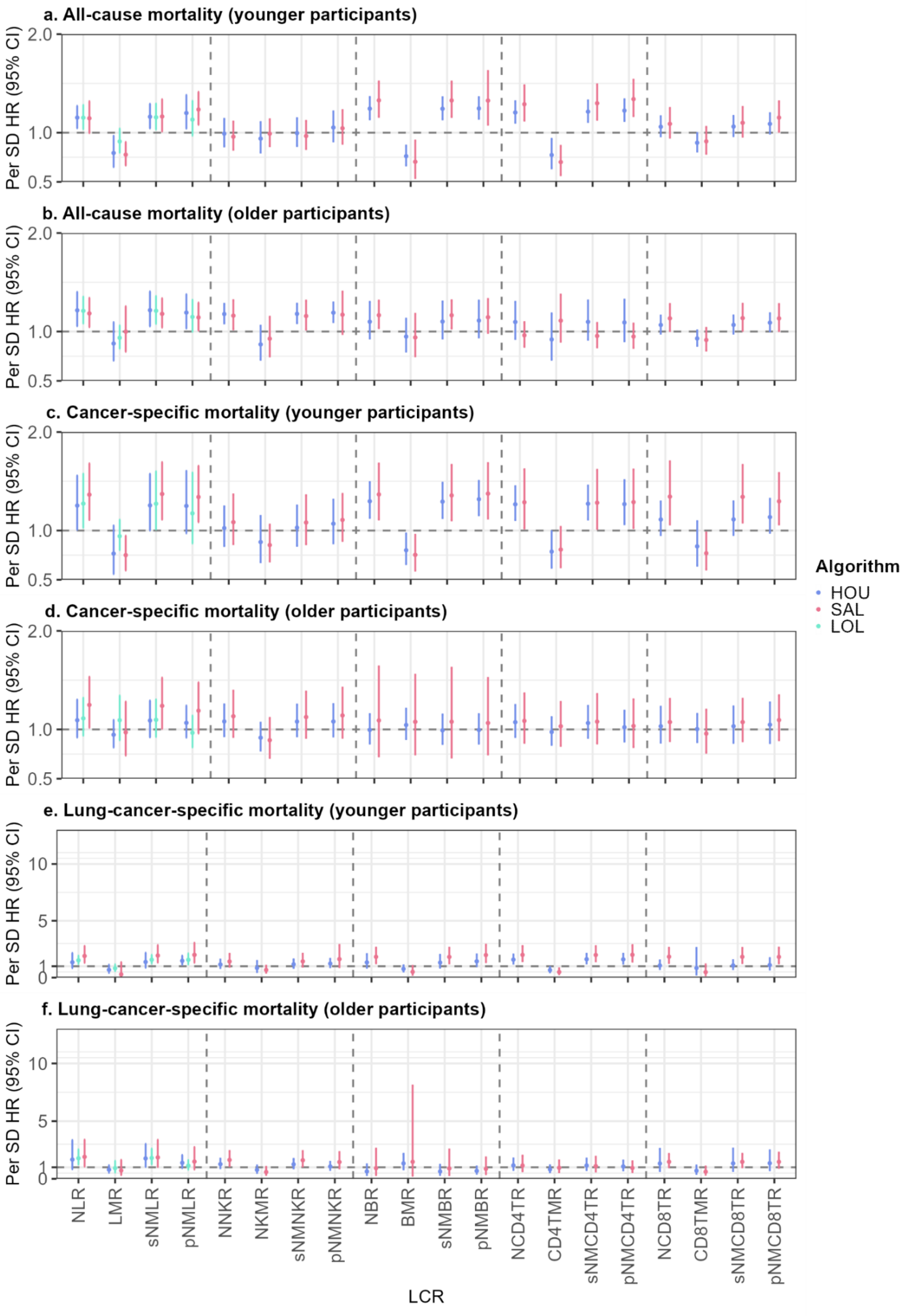

a. All-cause mortality (younger participants)
b. All-cause mortality (older participants)
c. Cancer-specific mortality (younger participants)
d. Cancer-specific mortality (older participants)
e. Lung-cancer-specific mortality (younger participants)
f. Lung-cancer-specific mortality (older participants)
Per SD HR (95% CI)
2.0
1.0
0.5
10
5
1
0
NLR
LMR
sNMLR
pNMLR
NNKR
NKMR
sNMNKR
pNMNKR
NBR
BMR
sNMBR
pNMBR
NCD4TR
CD4TMR
sNMCD4TR
pNMCD4TR
NCD8TR
CD8TMR
sNMCD8TR
pNMCD8TR
LCR
Algorithm
HOU
SAL
LOL

Figure S8. Association of DNAm-derived LCRs with mortality outcomes among younger and older participants.

The models were adjusted for sex, batch, smoking status, alcohol consumption, body mass index, educational level, physical activity, history of cardiovascular diseases, diabetes, and hypertension. Meta-analysis was performed for subsets I and II using the SAL algorithm and for all subsets using the HOU and LOL algorithms. Dot and error bars depict hazard ratios (HR) along with their 95% confidence intervals (CI).

Abbreviations: LCR, leukocyte composition ratio; HR, hazard ratio; CI, confidence interval; Per SD, per standard deviation increase; HOU, Housman's algorithm; SAL, Salas' algorithm; LOL, LOLIPOP algorithm; NLR, neutrophil-to-lymphocyte ratio; sNMLR, ratio of sum of neutrophil and monocytes divided by lymphocyte; pNMLR, ratio of product of neutrophil and monocytes divided by lymphocyte; LMR, lymphocyte-to-monocyte ratio; B, B cell; CD4T, $CD4^+$ T cell; CD8T, $CD8^+$ T cell; NK, natural killer cell.

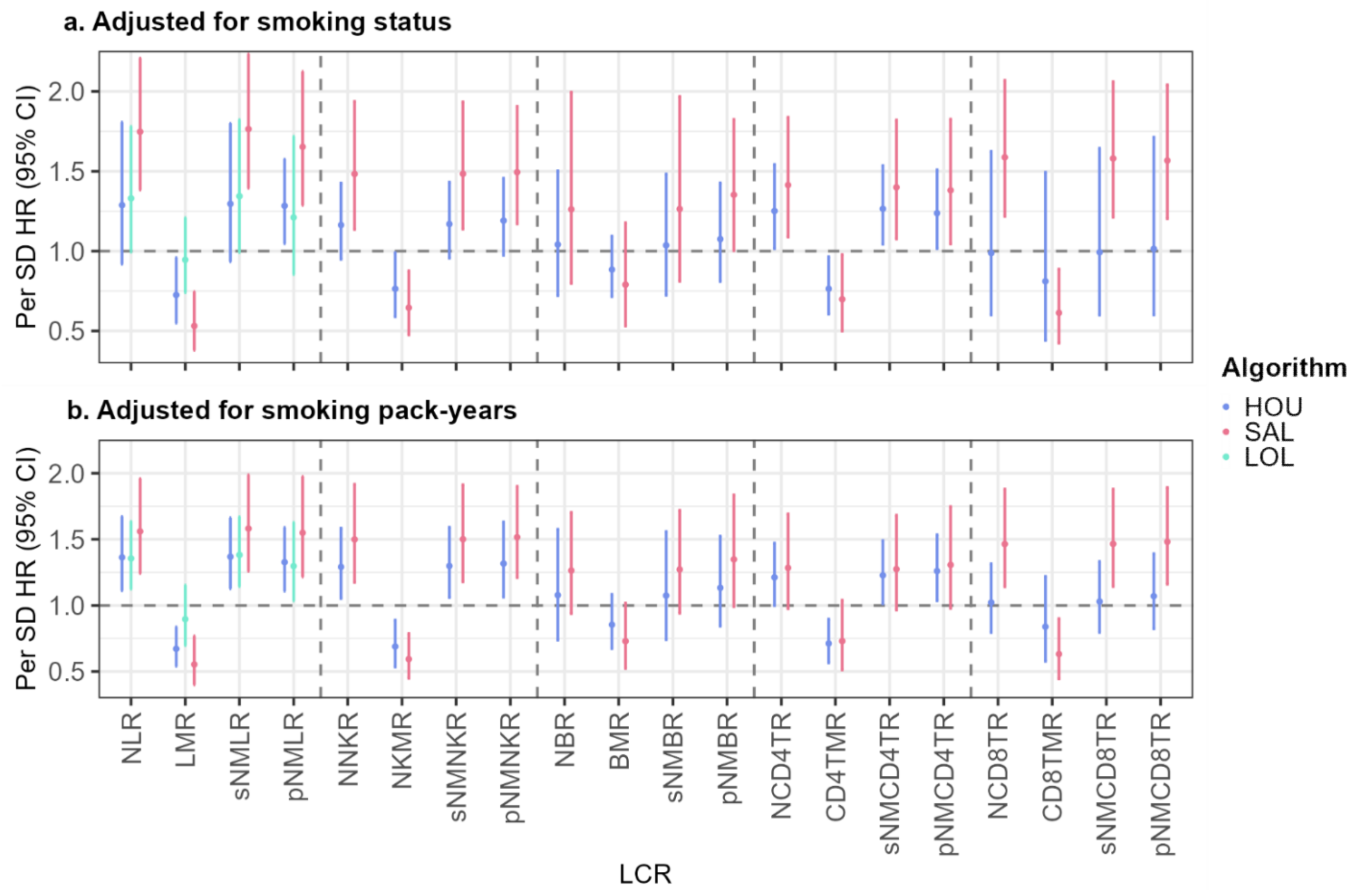

Figure S9. Association of DNAm-derived LCRs with mortality outcomes adjusted for smoking status or smoking pack-years in all subsets.

The models were adjusted for age, sex, batch, smoking status or smoking pack-years, alcohol consumption, body mass index, educational level, physical activity, history of cardiovascular diseases, diabetes, hypertension. Meta-analysis was performed for subsets I and II using the SAL algorithm and for all subsets using the HOU and LOL algorithms. Dot and error bars depict hazard ratios (HR) along with their 95% confidence intervals (CI).

Abbreviations: LCR, leukocyte composition ratio; HR, hazard ratio; CI, confidence interval; Per SD, per standard deviation increase; HOU, Housman's algorithm; SAL, Salas' algorithm; LOL, LOLIPOP algorithm; NLR, neutrophil-to-lymphocyte ratio; sNMLR, ratio of sum of neutrophil and monocytes divided by lymphocyte; pNMLR, ratio of product of neutrophil and monocytes divided by lymphocyte; LMR, lymphocyte-to-monocyte ratio; B, B cell; CD4T, $CD4^+$ T cell; CD8T, $CD8^+$ T cell; NK, natural killer cell.

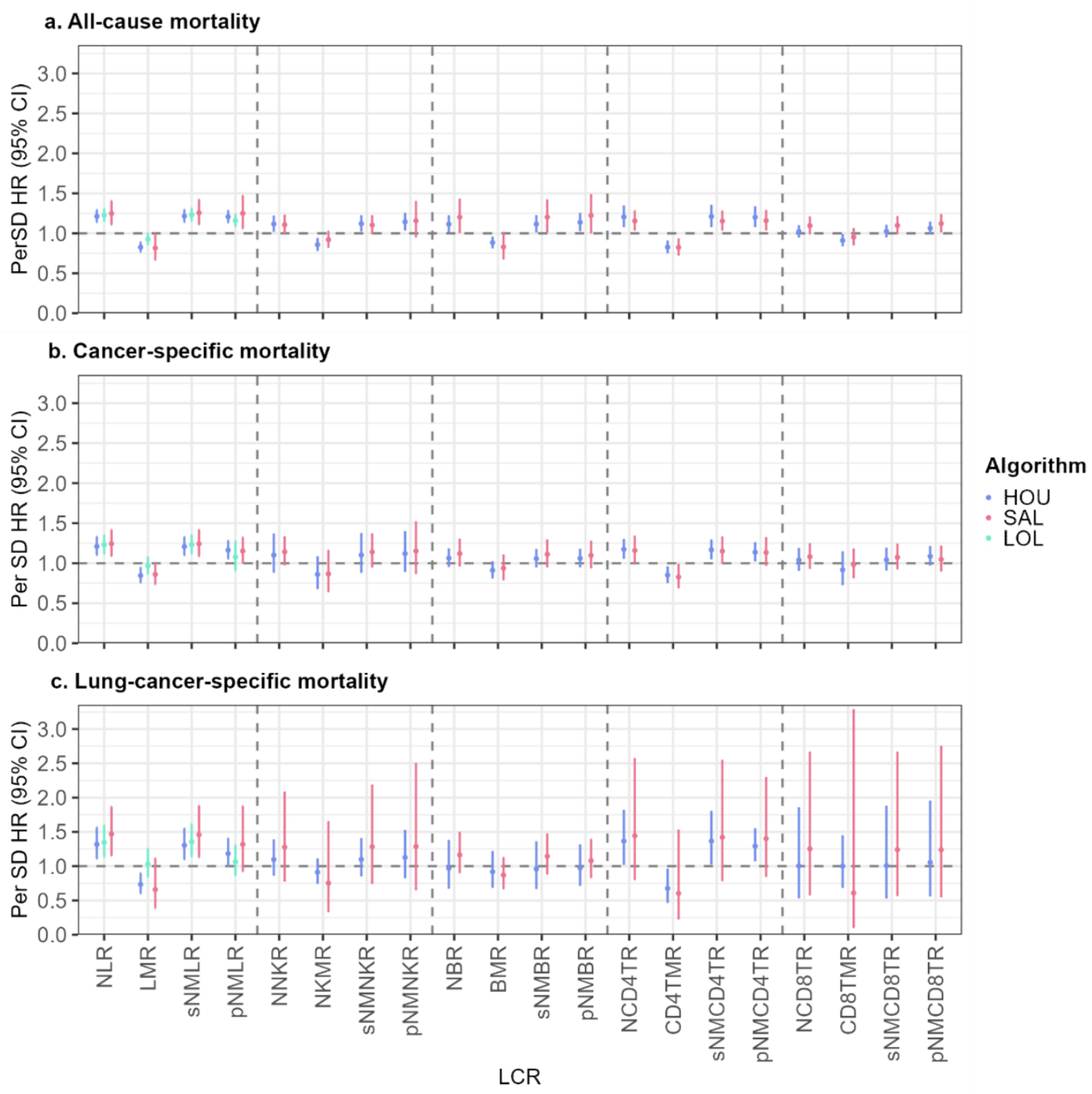

Figure S10. Association of DNAm-derived LCRs with 11-year follow-up mortality outcomes in all subsets.

The models were adjusted for age, sex, batch, smoking status, alcohol consumption, body mass index, educational level, physical activity, history of cardiovascular diseases, diabetes, and hypertension. Meta-analysis was performed for subsets I and II using the SAL algorithm and for all subsets using the HOU and LOL algorithms. Dot and error bars depict hazard ratios (HR) along with their 95% confidence intervals (CI).

Abbreviations: LCR, leukocyte composition ratio; HR, hazard ratio; CI, confidence interval; Per SD, per standard deviation increase; HOU, Housman's algorithm; SAL, Salas' algorithm; LOL, LOLIPOP algorithm; NLR, neutrophil-to-lymphocyte ratio; sNMLR, ratio of sum of neutrophil and monocytes divided by lymphocyte; pNMLR, ratio of product of neutrophil and monocytes divided by lymphocyte; LMR, lymphocyte-to-monocyte ratio; B, B cell; CD4T, $CD4^{+}$ T cell; CD8T, $CD8^{+}$ T cell; NK, natural killer cell.

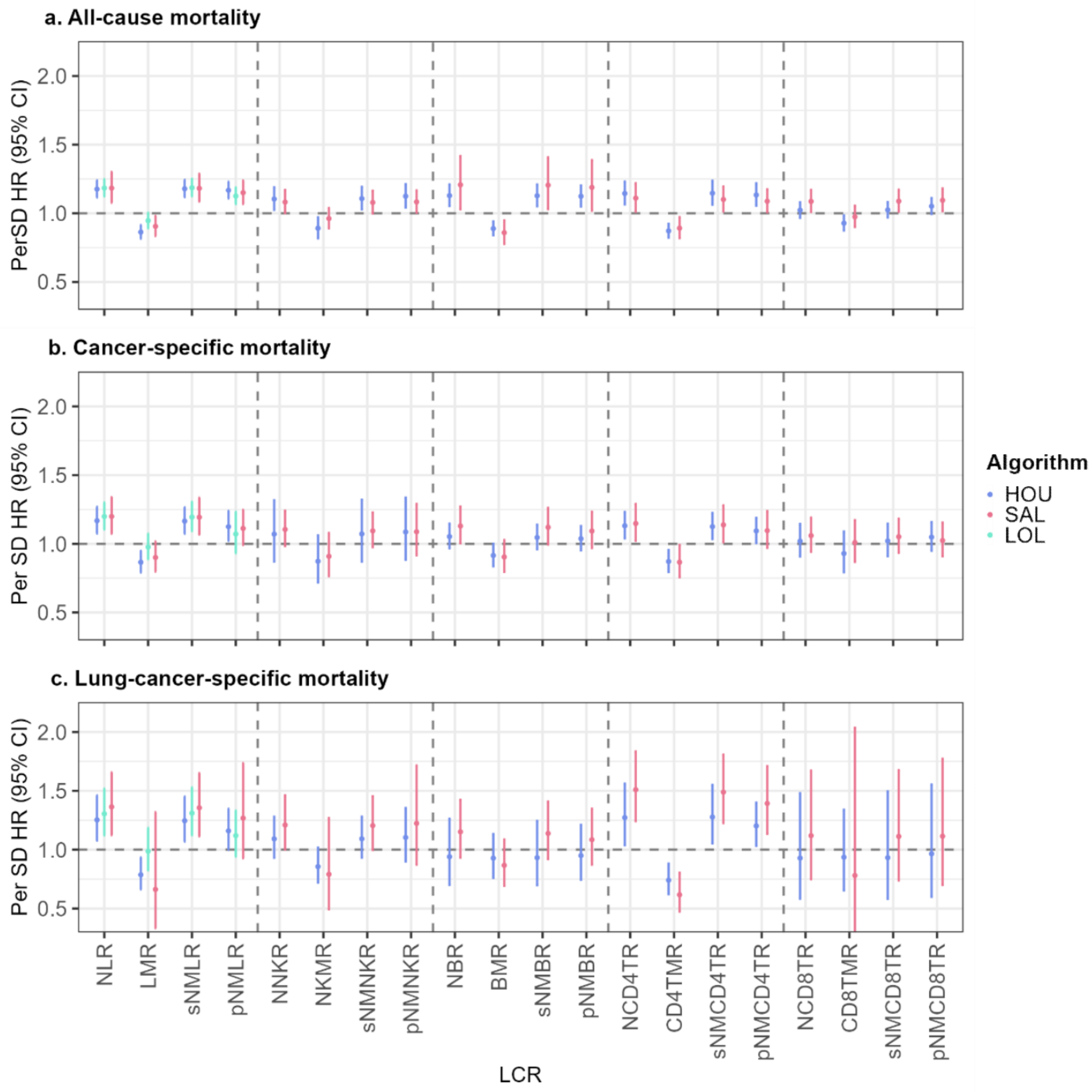

Figure S11. Association of DNAm-derived LCRs with 14-year follow-up mortality outcomes in all subsets.

The models were adjusted for age, sex, batch, smoking status, alcohol consumption, body mass index, educational level, physical activity, history of cardiovascular diseases, diabetes, and hypertension. Meta-analysis was performed for subsets I and II using the SAL algorithm and for all subsets using the HOU and LOL algorithms. Dot and error bars depict hazard ratios (HR) along with their 95% confidence intervals (CI).

Abbreviations: LCR, leukocyte composition ratio; HR, hazard ratio; CI, confidence interval; Per SD, per standard deviation increase; HOU, Housman's algorithm; SAL, Salas' algorithm; LOL, LOLIPOP algorithm; NLR, neutrophil-to-lymphocyte ratio; sNMLR, ratio of sum of neutrophil and monocytes divided by lymphocyte; pNMLR, ratio of product of neutrophil and monocytes divided by lymphocyte; LMR, lymphocyte-to-monocyte ratio; B, B cell; CD4T, $CD4^{+}$ T cell; CD8T, $CD8^{+}$ T cell; NK, natural killer cell.
