# Supplementary material for: Association of leukocyte composition ratios from blood methylation with cancer mortality outcomes
Source: Commun Med (Lond). 2025 Oct 1;5:411. doi: 10.1038/s43856-025-01132-x (PMC12488966; doi:10.1038/s43856-025-01132-x)
Supplement: Supplementary file 3 — Description of Additional Supplementary Files [file 43856_2025_1132_MOESM3_ESM.pdf]

Description of Additional Supplementary Files

Supplementary Data 1. Sensitivity analyses of dose-response relationship for association of DNAm-derived LCRs with all-cause mortality.

Supplementary Data 2. Sensitivity analyses of dose-response relationship for association of DNAm-derived LCRs with cancer-specific mortality

Supplementary Data 3. Sensitivity analyses of dose-response relationship for association of DNAm-derived LCRs with lung-cancer-specific mortality.
